# Cost-effectiveness of routine versus indicated antibiotic therapy in the management of severe wasting in children

**DOI:** 10.1186/s12962-022-00374-z

**Published:** 2022-08-03

**Authors:** Sheila Isanaka, Kevin Tang, Fatou Berthé, Rebecca F. Grais, Ankur Pandya

**Affiliations:** 1 Department of Research, Epicentre, 14-34 avenue Jean Juarès, 75019 Paris, France; 2 Department of Nutrition, Harvard T.H. Chan School of Public Health, Boston, MA USA; 3 Department of Population Health, London School of Hygiene & Tropical Medicine, London, UK; 4 Epicentre Niger, Niamey, Niger; 5 Department of Health Policy and Management, Harvard T.H. Chan School of Public Health, Boston, MA USA

**Keywords:** Cost-effectiveness, Antibiotic therapy, Wasting, Severe acute malnutrition, Niger

## Abstract

**Background:**

In the outpatient management of severe wasting, routine antibiotic therapy is recommended for all children upon admission regardless of whether clinical signs of infection are present. Indicated antibiotic therapy, where antibiotics are provided only upon presentation of clinical signs of infection, may be considered for its potential to allow for more prudent antibiotic use and greater program coverage, reducing the risk of antibiotic resistance as well as costs and logistical burdens associated with treatment. We therefore conducted a cost-effectiveness analysis to measure the effects of indicated antibiotic therapy compared to routine antibiotic therapy in terms of incremental cost-per-life-year saved in Niger.

**Methods:**

We used a cohort model to conduct a cost-effectiveness analysis from a healthcare system perspective to project and weigh the lifetime discounted costs and effects of indicated antibiotic therapy compared to routine antibiotic therapy in the treatment of uncomplicated severe wasting in children in Niger. We calculated incremental cost-effectiveness ratios (ICERs) in terms of treatment-related healthcare costs per discounted life-years saved (LYS), and conducted program coverage scenario and sensitivity analyses to assess model uncertainty.

**Results:**

The ICER for indicated antibiotic therapy compared to routine antibiotic therapy was $8.5/LYS, which is under the cost-effectiveness threshold for Niger. The probability of the indicated strategy being optimal was 76.1% when program coverage was equal to coverage associated with routine therapy but was 100% likely to be optimal in probabilistic sensitivity analysis scenarios where indicated program coverage improved 5 percentage points.

**Conclusions:**

Indicated antibiotic therapy likely represents a cost-effective strategy, particularly if indicated treatment can result in expanded coverage. With the risk of increasing antibiotic resistance worldwide, antibiotic stewardship and simplified treatment protocols for severe wasting using indicated antibiotic therapy may represent good value for money in some low risk populations.

**Supplementary Information:**

The online version contains supplementary material available at 10.1186/s12962-022-00374-z.

## Background

Antibiotic resistance can cause infections that can be difficult and costly to treat and is a problem of growing concern [1]. Antibiotic stewardship, including systematic efforts to improve how antibiotics are prescribed, is an essential element in the global approach to manage infections and combat antibiotic resistance.

Severe wasting affects at least 14 million children under 5 years of age and contributes to more than half a million deaths each year [2, 3]. In the outpatient management of severe wasting, the World Health Organization (WHO) recommends broad spectrum oral antibiotics for all children upon admission regardless of whether clinical signs of infection are present [4]. The rationale for routine antibiotic therapy to all children comes from the view that malnourished children may not show signs of clinical infection [5]. Historic clinical trials have shown antibiotics to decrease mortality among malnourished children with clinical complications treated in hospital [6, 7]. However, evidence supporting routine antibiotic therapy among children without clinical complications treated in outpatient settings today is weak [4], and conflicting results from recent randomized trials suggest that the clinical benefit of routine antibiotic therapy may vary due to context-specific factors and be limited to high risk populations [8–10].

In 2014, we conducted a randomized, placebo-controlled trial among severely wasted children in Niger to assess the effect of indicated vs. routine antibiotic therapy on nutritional recovery (ClinicalTrials.gov number, NCT01613547). Indicated antibiotic therapy, where treatment was provided upon presentation with clinical signs of infection, was evaluated given its potential to allow for more prudent antibiotic use that could also reduce costs and logistical burdens associated with treatment. We found routine antibiotic therapy did not impact the likelihood of sustained nutritional recovery (risk ratio, RR: 0.95, 95% confidence interval [CI] 0.86 to 1.05) or the risk of transfer to inpatient care up to 12 weeks, compared to indicated antibiotic therapy (RR = 0.97, 95% CI 0.84, 1.13) [9].

Global guidance on routine antibiotic therapy in the treatment of severe wasting should weigh both individual and public health risks and benefits, including the emergence of antibiotic resistance, possible individual side effects and cost and logistical considerations that enable scaling up within national nutritional programs. The optimal strategy would be associated with the best clinical outcome at the lowest price with the least selective pressure for antibiotic resistance. Assessing the cost-effectiveness of antibiotic therapy in the treatment of severe wasting aims to explore whether routine antibiotic treatment makes a sufficient contribution to health to justify its costs. To broaden the available evidence to inform use of routine antibiotics among malnourished children, we specifically conducted a cost-effectiveness analysis to measure the effects of indicated antibiotic therapy compared to routine antibiotic therapy in terms of incremental cost-per-life-year saved in Niger and formally evaluate the impact of program costs and increased coverage scenarios not captured in the trial but are nonetheless influential for decision-making.

## Methods

### Overview

**Fig. 1:**
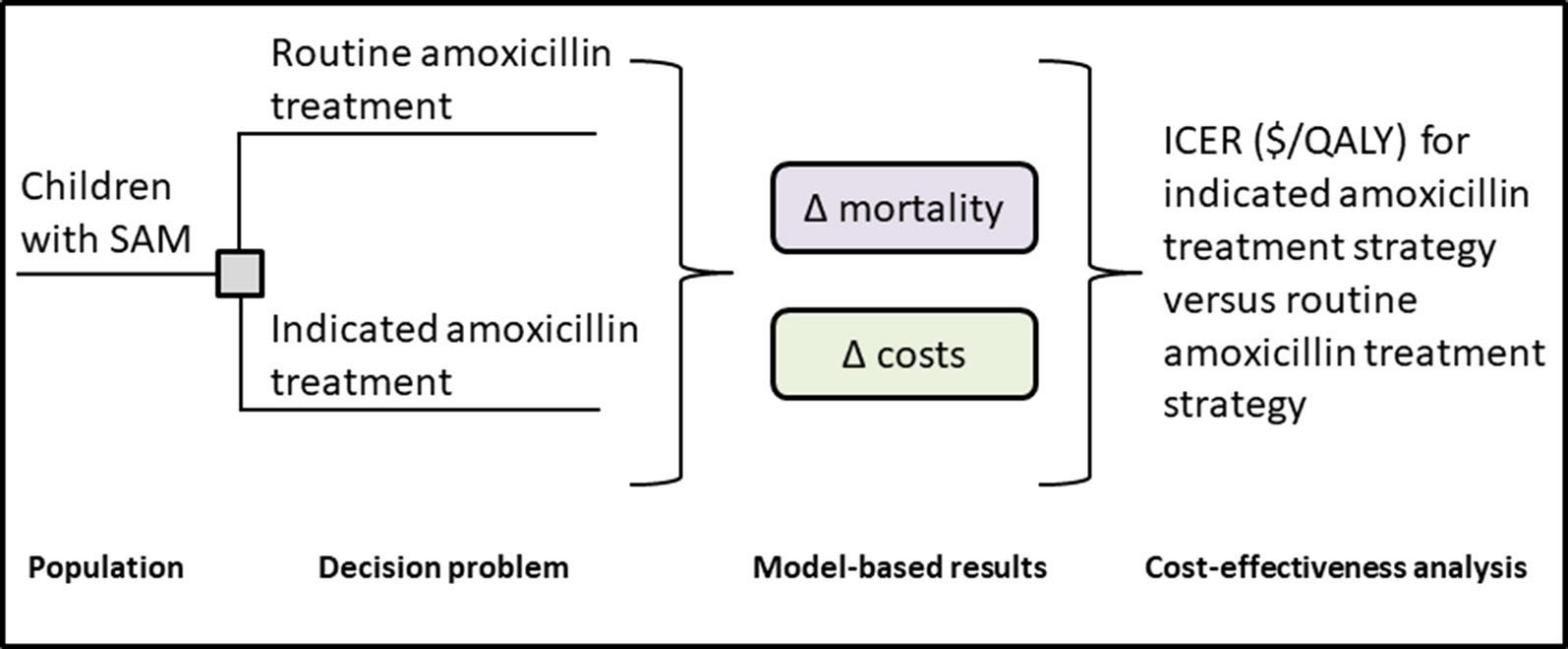
Conceptual diagram of the cost effectiveness analysis. Individuals enter the simulation model and are assigned to one of two strategies for antibiotic therapy. The model estimates the impact of indicated vs. routine antibiotic therapy on mortality and cost outcomes. The tradeoffs between life years saved (LYS) and costs are evaluated by calculating an incremental cost-effectiveness ratio (ICER) for indicated antibiotic therapy compared to routine antibiotic therapy

We developed a computer-based simulation model to estimate life expectancy and intervention-related costs of indicated vs. routine antibiotic therapy in the treatment of uncomplicated severe wasting in children in Niger. We compared the tradeoff between lifetime discounted health effects, quantified using life years saved (LYS), and treatment-related healthcare costs using incremental cost-effectiveness analysis from a healthcare system perspective. The simulation model discounted health effects and costs using an annual rate of 3.5%. An overview of our methodology is shown in Fig. 1. The parent trial protocol was approved by the Comité Consultatif National d’Éthique, Niger and the Comité de Protection des Personnes, Île-de-France XI, Paris.

### Simulation model and population

Our model was structured for three time periods to match our data sources: (1) admission for outpatient severe wasting treatment to 12 weeks; (2) 12 weeks to 1 year; and (3) 1 year until death. The model population was determined by the proportion of children under 5 years of age [11] and the prevalence of severe wasting in Maradi, Niger [12]. Our model projected acute and lifetime outcomes for a population of 100,000, i.e., a cohort model as opposed to an individual-level microsimulation, the size of a typical rural health district in Niger (7764 of whom would be under 5 years of age with severe wasting). Children with severe wasting started the model with an age of 17 months and were modeled whether or not they were covered by the program. Table 1 shows all model input parameter base case values and data sources.

**Table 1:** Model variables with base case values and ranges used in one-way sensitivity analysis

Variable	Base case value	Sensitivity analysis range	Probability distribution for sensitivity analyses	Source(s)
Population demographics				
Proportion of population under 5 years	20.4%	15.3–25.5%	Beta	11
Prevalence of severe wasting in children under 5	5.3%	4.0–6.6%	Beta	^12^
Severe wasting incidence correction factor	7.2	5.4–9.0	Normal	^34^
Severe wasting treatment point coverage	19.6%	14.8–24.5%	Beta	^16^
Natural history				
Annual background mortality rate for non-wasted children 1–5 year in Niger	2.2%	1.7–2.8%	n/a	^11, 17^
Hazard ratio of mortality among children with untreated moderate wasting	3.4	2.6–4.3	Lognormal	^2^
Hazard ratio of mortality among children with untreated severe wasting	11.6	8.7–14.5	Lognormal	^2^
Duration of untreated severe wasting episode (weeks)	20.2	15.2–25.3	Gamma	^35^
Routine treatment outcomes at 12 weeks				
Number recovered after severe wasting treatment	733	550–920	Dirichlet	^9, 10, 36, 37^
Number of non-responders to severe wasting treatment	63	47–79	Dirichlet	^36^
Number defaulting from severe wasting treatment	12	9–15	Dirichlet	^36^
Number transferring to inpatient care	370	278–463	Dirichlet	^9, 36^
Number of deaths after transfer to inpatient care during treatment	5	4–6	Dirichlet	^34^
Number of deaths during severe wasting treatment	21	16–58	Dirichlet	^9, 10, 36^
Average days to recovery	28.3	21.2–35.4	Gamma	^9, 36^
Average days to default	24.3	18.2–30.4	Gamma	^36^
Average days to death	28.9	21.7–36.1	Gamma	^9, 36^
Average days to transfer	24.8	18.6–31.0	Gamma	^9, 36^
Indicated treatment outcomes at 12 weeks				
Number recovered after severe wasting treatment	700	525–875	Dirichlet	^9, 36^
Number of non-responders to severe wasting treatment	47	35–59	Dirichlet	^36^
Number defaulting from severe wasting treatment	9	7–11	Dirichlet	^36^
Number transferring to inpatient care	427	320–534	Dirichlet	^9, 36^
Number of deaths after transfer to inpatient care during treatment	5	4–6	Dirichlet	^34^
Number of deaths during severe wasting treatment	17	13–21	Dirichlet	^9, 36^
Average days to recovery	30.2	22.7–37.8	Gamma	^9, 36^
Average days to default	24.9	18.7–31.1	Gamma	^36^
Average days to death	17.5	13.1–21.9	Gamma	^9, 36^
Average days to transfer	24.1	18.1–30.1	Gamma	^9, 36^

n/a stands for not applicable, i.e. not used in probabilistic sensitivity analysis due to lack of data needed to inform probability distribution

### Mortality effects

Mortality risks were considered separately for the three time periods of the model (Additional file 1: Fig. S1). Mortality risks in the first time period (treatment admission to 12 weeks) were based on the arm-specific results from the parent randomized controlled trial designed to assess the impact of routine amoxicillin prescription on nutritional recovery [9]. Per the parent trial, 12-week treatment outcomes were divided into six acute outcomes: (1) nutritional recovery with no relapse; (2) nutritional recovery with relapse; (3) non-response after 8 weeks; (4) transfer to inpatient care; (5) default; and (6) death. The distribution of these outcomes for the routine and indicated antibiotic treatment strategies were taken directly from the parent trial data.

Mortality risks in the rest of the first year (weeks 13–52) were based on whether or not the child experienced a second episode of severe wasting after recovery from the index case (e.g., relapse). The risk of relapse was assumed to be 10.5% [13], with the associated mortality risk of the relapse event assumed to be equal to that of the first episode. Mortality during the first year without relapse and after the first year was based on regional mortality tables for non-wasted children [11] and a hazard ratio of 1.2 for mortality after recovery [14]. Additional file 1: Fig. S1 shows how these risks are applied in the model during each time period (admission to 3 weeks, 4–12 weeks, ≥ 13 weeks).

### Intervention costs (2017 USD)

Intervention costs considered all treatment-related healthcare expenditures from admission to 1 year, including inpatient care associated with transfer or non-response during treatment and relapse up to 1 year. Unit costs for expenditure within four major cost categories of treatment (personnel; therapeutic food; medical supplies; and infrastructure and logistical support) were estimated using a micro-costing analysis of the treatment of severe wasting in Niger [15]. Marginal costs of therapeutic food and medical supplies varied by the number of children enrolled in a treatment strategy and length of stay by the six acute outcomes observed in the parent trial (Table 1 and Additional file 2: Table S1). Costs of personnel, infrastructure and logistical support were fixed, assuming ten outpatient health centers, one inpatient stabilization center and a community-based screening team for the model population of 100,000 in the typical rural health district (Additional file 2: Table S2).

### Cost-effectiveness and sensitivity analyses

We used conventional incremental cost-effectiveness analysis methods to calculate an incremental cost-effectiveness ratio (ICER) for indicated antibiotic therapy compared to routine antibiotic therapy. Incremental effectiveness was defined using LYS as the difference in projected discounted life expectancy between the indicated versus routine antibiotic strategies. We used the per capita Gross Domestic Product (GDP) for Niger ($378 [16]) as our cost-effectiveness threshold given the lack of a relevant country-specific or region-specific opportunity cost-based threshold. We assumed 19.6% nutritional program coverage for both the routine and indicated treatment strategies in our base case analysis. [16] For children not covered by the program, mortality risks were calculated by applying the hazard ratio for untreated severe wasting (11.6 [2]) to the annual background mortality rate for non-wasted children 1-5 year in Niger (2.2% [11, 17]). In the model, the impact of program coverage depended on whether the routine or indicated antibiotic strategy was being evaluated, with the model applying arm-specific mortality effects conditional on coverage. We hypothesized that indicated antibiotic therapy could support increased program coverage if treatment with indicated antibiotic therapy were shifted to community health workers at the village-level reducing potential barriers to access. We therefore varied coverage levels in the indicated treatment strategy to 25%, 30%, 50%, and 100% in coverage scenario analyses. We assumed constant per child treatment costs as program coverage increased due to the lack of quantitative estimates of non-linear cost-coverage functions [18]. Due to limited data on how treatment costs increase non-linearly with program coverage, we conducted a two-way sensitivity analysis in which we varied cost per child and program coverage for the indicated treatment strategy given the joint importance of these variables.

We limited the analytical time horizon to the 12-week trial period in sensitivity analysis. We further varied all model inputs through upper and lower bounds in one-way sensitivity analyses and performed a probabilistic sensitivity analysis by drawing 1000 model input parameters from the probability distributions shown in Table 1 [18]. We based these distributions on the uncertainty of the data source for each of these inputs (e.g., 95% confidence intervals around the point estimates) and logical constraints (e.g., beta distributions for probability values, which must have values between 0 and 1). We used the Dirichlet distribution, a multivariate generalization of the beta distribution, [19] for the 12-week treatment outcomes given the six possible mutually exclusive outcomes described above. The model projected LYS and incremental cost outcomes for each of the 1000 probabilistic sensitivity analysis iterations, which were shown in a scatterplot to represent overall model uncertainty.

## Results

### Base case results

From a population size of 100,000, 20,430 individuals were estimated to be under 5 years of age, with 7764 total cases of severe wasting expected over a 12-month period. For this cohort of children with severe wasting, the model projected 395,043 undiscounted life years, 147,385 discounted life years, and $1,862,478 in treatment costs ($239.80 per child treated) with routine antibiotic therapy. The same results were 395,480 undiscounted life years, 147,552 discounted life years, and $1,863,897 in treatment costs ($240.10 per child treated) with indicated antibiotic therapy. The ICER (using discounted LYS) for indicated antibiotic therapy was $8.5/LYS compared to the routine antibiotic therapy (Table 2). Greater life years but higher costs with indicated antibiotic therapy compared to routine antibiotic therapy in the base case were, respectively, driven by the lower risk of death up to 12 weeks from admission (1.4% vs 1.8%) and greater time to recovery (30.2 days vs 28.3 days) observed in the parent trial.

**Table 2:** Lifetime per-person life years, costs ($), and incremental cost-effectiveness ratios for a population size of 100,000 (7,764 total cases of severe acute malnutrition)

Strategy	Total children treated	Undiscounted life years	Incremental discounted LYS^a^	Costs^a^	ICER	Probability dominant strategy^b^ (%)	Probability optimal strategy^c^ (%)
19.6% program coverage for indicated and routine antibiotic therapy							
Routine antibiotic therapy	1522	395,043	Reference	$1,862,478	Reference	10.8	23.9
Indicated antibiotic therapy	1522	395,480	167	$1,863,897	$8.5/LYS	37.5	76.1
25% program coverage for indicated antibiotic therapy; 19.6% coverage for routine antibiotic therapy							
Routine antibiotic therapy	1522	395,043	Reference	$1,862,478	Reference	0.0	0.0
Indicated antibiotic therapy	1941	400,110	1,928	$1,887,923	$13.2 /LYS	10.2	100.0
30% program coverage for indicated antibiotic therapy; 19.6% coverage for routine antibiotic therapy							
Routine antibiotic therapy	1522	395,043	Reference	$1,862,478	Reference	0.0	0.0
Indicated antibiotic therapy	2329	404,397	3,560	$1,910,169	$13.4/LYS	1.9	100.0
50% program coverage for indicated antibiotic therapy; 19.6% coverage for routine antibiotic therapy							
Routine antibiotic therapy	1522	395,043	Reference	$1,862,478	Reference	0.0	0.0
Indicated antibiotic therapy	3882	421,544	10,085	$1,999,152	$13.6/LYS	0.0	100.0
100% program coverage for indicated antibiotic therapy; 19.6% coverage for routine antibiotic therapy							
Routine antibiotic therapy	1522	395,043	Reference	$1,862,478	Reference	0.0	0.0
Indicated antibiotic therapy	7764	464,411	26,399	$2,221,611	$13.6/LYS	0.0	100.0

^a^Discounted at an annual rate of 3.5%

^b^Based on probabilistic sensitivity analysis, where “dominant” means higher LYS and lower costs compared to the competing strategy

^c^Based on probabilistic sensitivity analysis, where “optimal” is based on a cost-effectiveness threshold of $378/LYS for Niger

### One-way sensitivity analyses

Additional file 2: Table S3 shows the results of the one-way sensitivity analysis for all model parameters shown in Table 1. The ICER for indicated antibiotic therapy compared to routine antibiotic therapy was most sensitive to the risk of death during treatment and time until recovery, but ranged from the indicated strategy strongly dominating the routine strategy (i.e., the indicated strategy had higher LYS and lower costs) to $65/LYS, suggesting model results were robust to deterministic changes in individual parameters.

### Coverage scenario and probabilistic sensitivity analyses

**Fig. 2:**
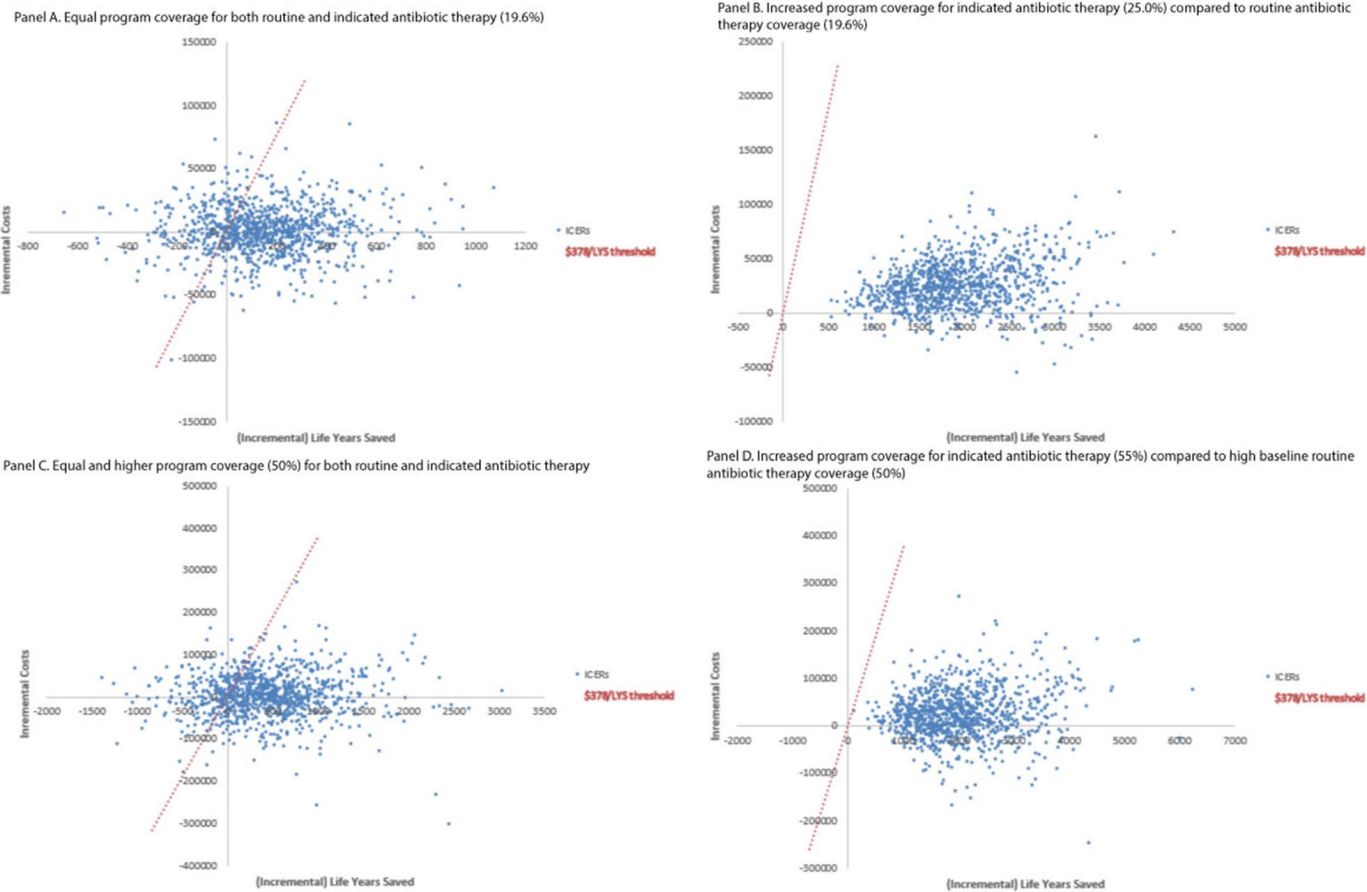
Scatterplot for the probabilistic sensitivity analysis (PSA) for four coverage scenarios (both routine and indicated strategies with 19.6% coverage in **A**; routine strategy with 19.6% coverage and indicated strategy with 25.0% coverage in **B**; both routine and indicated strategies with 50.0% coverage in **C**; and routine strategy with 50.0% coverage and indicated strategy with 55.0% coverage in **D**

Table 2 shows the results for model scenarios assuming equal program coverage levels of 19.6% for both strategies (base case analysis), and alternative scenarios where the indicated treatment strategy achieved higher program coverage levels (25%, 30%, 50%, and 100%) and treatment cost per child was held constant for each level of program coverage. The ICER for the indicated therapy strategy compared to the routine antibiotic therapy strategy remained under the cost-effectiveness threshold of $378/LYS (e.g., one times per capita GDP for Niger) in all coverage scenarios. The probability of the indicated strategy being optimal (at a cost-effectiveness threshold of $378/LYS) was 76.1% when coverage was equal to routine coverage (19.6%) and 100% in scenarios where average indicated program coverage increased from 19.6 to 25% or more (holding average routine coverage at 19.6%, Fig. 2A and B). The probability of the indicated strategy being optimal did not substantially change (76.1% vs. 78.8%) in a coverage scenario with equal higher coverage (50%, Fig. 2C) and was again 100% with a five percentage point increase in coverage with the indicated strategy (55.0%) compared to routine strategy with high baseline coverage (Fig. 2D). The high probability of the indicated strategy being optimal when coverage was expanded by at least 5 percentage points was consistent across all levels of baseline coverage (Additional file 2: Table S4). When the analytic time horizon was limited to 12 weeks, the indicated treatment strategy was optimal in 51% of iterations.

**Fig. 3:**
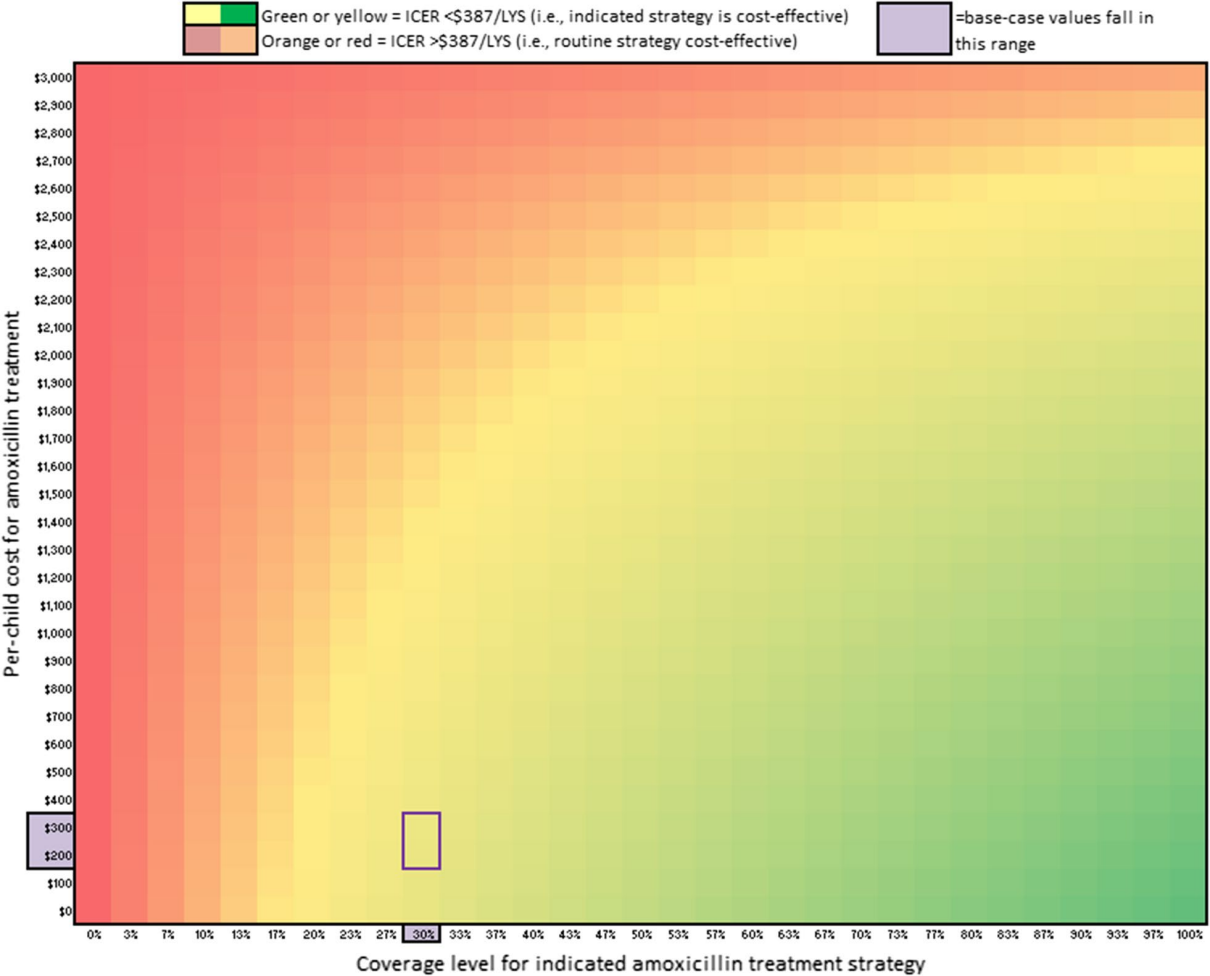
Two-way sensitivity analysis showing the optimal strategy for different combinations of costs per child treated and coverage. Indicated antibiotic therapy is optimal in the green–yellow region, which includes the base case result outlined with a box. Routine antibiotic therapy is optimal in the red–orange region

Figure 3 shows the results from the 2-way sensitivity analysis varying cost per child treated and coverage for the indicated treatment strategy: the ICER for the indicated treatment strategy was only above the $378/LYS cost-effectiveness threshold at very high per child treatment cost (for example, greater than $1400 per child with 30% indicated program coverage or greater than $2200 per child with 50% indicated program coverage), while by comparison, the base case cost of treatment with indicated therapy was $240 per child treated.

## Discussion

Here we present the first cost-effectiveness analysis to weigh the gains in life expectancy against treatment-related costs of indicated versus routine antibiotic therapy in the outpatient treatment of severe wasting. Indicated antibiotic therapy was not cost-saving in most probabilistic simulations, but likely cost-effective when compared to routine antibiotic therapy in terms of incremental cost-per-life-year-saved, particularly when associated with increased program coverage.

The debate surrounding the use of antibiotics is not straightforward and both clinical and public health risks and benefits must be considered. To date, clinical evidence to support the use of routine antibiotic therapy in the outpatient treatment of severe wasting is limited. Conflicting results from recent randomized trials suggest that the efficacy of antibiotic therapy may vary due to context-specific factors such as the baseline risk of mortality and that benefit may be limited to high risk populations [8–10]. Without an increase in program coverage resulting from use of the indicated antibiotic therapy strategy, there was some uncertainty in our model regarding which strategy was optimal, reflecting in part the lack of a statistically significant treatment effect from the parent trial. However, with a small increase in program coverage of just over five percentage points, the indicated strategy was optimal in 100% of probabilistic iterations. Future experience on whether such an increase in program coverage with indicated antibiotic therapy is plausible would reduce the uncertainty regarding which strategy is the more cost-effective use of limited health care resources.

In settings with a strong health system, indicated antibiotic therapy could save precious healthcare resources, decrease the risk of unnecessary side effects and adverse effects, and may delay the development of antibiotic resistance through antibiotic stewardship. The development of antibiotic resistance would depend on a number of factors, including the prevalence and composition of pre-existing resistance in the community, adherence to antibiotic regimens, and the prevalence of severe wasting. The effect of routine antibiotic therapy on the emergence and transmission of antibiotic resistance has been shown in this study population [20]. The risks and costs potentially resulting from the development of antibiotic resistance associated with routine antibiotic therapy in the management of severe wasting were not considered in this analysis due to uncertainty of parameter estimates but should not be ignored. With the WHO giving a central role to amoxicillin in its essential medicines list [21], Mass administration should be carefully considered to ensure that essential antibiotics remain effective to treat infection.

In addition, routine antibiotic therapy has important theoretical implications for program costs and coverage in the outpatient management of severe wasting. The financial costs of a course of routine antibiotics are not relatively large (USD 1.08 per child weighing 6.5 kg [22]). However, in some settings, providing routine antibiotics can require more specialized medical personnel at health structures and a reliable supply chain management, which may limit the scale of programs and the number of children reached through national initiatives in resource limited settings. The use of indicated antibiotic therapy has the potential to allow for greater flexibility to provide nutritional treatment at the village-level through community health workers. Such decentralization may reduce barriers associated with caregiver travel and opportunity costs associated with health center visits to improve program coverage [23, 24]. In one-way sensitivity analyses that allowed for a wide range of values for the risk of death, the risk of transfer to inpatient care, and time to recovery, indicated antibiotic therapy remained cost effective.

One limitation of our analysis was that we modeled linear cost increases as coverage increased, although it is quite possible that costs increase non-linearly as program coverage is expanded to remote areas. Though data was lacking to define a non-linear cost-coverage function over the range of program coverage levels, our two-way sensitivity analyses showed that indicated antibiotic therapy was cost effective for any reasonable cost function. The threshold where indicated antibiotic therapy would no longer be cost effective (where Fig. 2 changes from yellow to orange) was found at implausibly high levels of treatment costs ($2000 costs per child treated compared to reported costs of $135–442 [25–29]), providing confidence that this issue would not change our main conclusions. Further, this analysis did not model the costs of antibiotic resistance that would render essential antibiotics ineffective to treat infection, and therefore presents a conservative cost-effectiveness estimate of indicated antibiotic therapy. This analysis considered all treatment-related costs from a healthcare system perspective but did not consider household costs associated with treatment. Specifically, more frequent hospitalization associated with indicated antibiotic therapy could bear additional household costs that were not accounted for in the model considering costs from a healthcare system perspective only. We also used a GDP-based cost-effectiveness threshold, which is less optimal than an empirically estimated opportunity cost-based cost-effectiveness threshold. While there currently is no such opportunity cost-based threshold for Niger [30], most results were not sensitive to the cost-effectiveness threshold. Finally, it was difficult to draw conclusions within the short trial period: the indicated antibiotic strategy was cost-effective in 51% of probabilistic sensitivity analysis iterations (equivalent to a null result) when restricting the analysis to a 12-week analytic time horizon. However, best modeling practice suggests using an analytic time horizon that is long enough to capture all relevant health and cost outcomes [31], which for interventions that affect mortality such as severe wasting is a lifetime analytic time horizon. Under the lifetime analytic time horizon of the present analysis, we show indicated antibiotic strategy was optimal in > 75% of probabilistic sensitivity analysis iterations.

Outpatient management for severe wasting has been adopted in over 70 countries [32] and all protocols include the provision of routine antibiotics for the more than 14 million children suffering from severe wasting [33]. Where it can save lives such as in high risk populations, routine antibiotic use should remain a part of clinical protocols to reduce mortality. However, with the risk of increasing antibiotic resistance worldwide and conflicting data on the efficacy routine antibiotics in diverse programmatic settings, decision analysis is warranted to provide an additional perspective to inform guidance. It is possible that simplified treatment protocols using indicated antibiotic therapy may be appropriate, and even preferable, in low risk populations with a strong health system. While there is likely no single solution, guidance that allows treatment protocols to be adapted and simplified in specific contexts while maintaining individual effectiveness, protecting public health safety, and supporting increased program coverage should be prioritized.

## Conclusions

The present analysis used unique trial-based data and cohort models to examine life expectancy and treatment-related costs with extensive scenario and sensitivity analyses and adds new evidence to inform global guidance and practice on the use of routine antibiotics in treatment of severe wasting. While routine antibiotic therapy may provide clinical benefit in high risk populations, indicated antibiotic therapy represented a cost-effective strategy, particularly with program coverage expansion of 5 more percentage points. This information provides support for indicated antibiotic therapy in some settings, although future research on whether the indicated strategy could result in increased program coverage and the shape of the cost function from coverage expansions is warranted.

## Supplementary Information

**Additional file 1**: **Figure S1.** Schematic for modelled risk of mortality over time by treatment outcome.

**Additional file 2**: **Table S1.** Costs of therapeutic food and medical supplies per child-outcome. **Table S2.** Costs of personnel, infrastructure and logistical support per fixed cost category. **Table S3.** One-way sensitivity analysis results. **Table S4.** Probabilistic sensitivity analysis results with varying levels of coverage for the routine and indicated treatment strategies.

## Data Availability

The de-identified dataset, code book, and analytic code supporting this research will be made available from the corresponding author, following a reasonable submitted request.

## Abbreviations

CI: Confidence interval
GDP: Gross domestic product
ICER: Incremental cost-effectiveness ratios
LYS: Life-years saved
WHO: World Health Organization
