# Supplementary material for: Cost-effectiveness of routine versus indicated antibiotic therapy in the management of severe wasting in children
Source: Cost Eff Resour Alloc. 2022 Aug 3;20:38. doi: 10.1186/s12962-022-00374-z (PMC9351197; doi:10.1186/s12962-022-00374-z)
Supplement: Supplementary file 1 — Additional file 1: Figure S1. Schematic for modelled risk of mortality over time by treatment outcome. [file 12962_2022_374_MOESM1_ESM.pdf]

Figure S1. Schematic for modelled risk of mortality over time by treatment outcome

RISK OF DEATH DURING AND AFTER TREATMENT

**Observation period**	Parent trial follow-up			Post-recovery (a)									
Months since admission	1	2	3	4	5	6	7	8	9	10	11	12	13+
Nutrition recovery (no relapse)													Life tables (e)
Nutrition recovery (relapse)						Relapse (b)							Life tables (e)
Non-response after 8 wk													Life tables (e)
Default	(c)	Post-default period (d)											Life tables (e)
Transfer to inpatient care		(f)											Life tables (e)
Death													

(a) Post-recovery mortality based on Niger baseline mortality with adjustment for mortality after recovery (hazard ratio=1.2)
(b) Relapse mortality based on baseline Niger mortality with adjustment for treated severe wasting
(c) Acute default mortality assumed to be 50% in first 3 weeks
(d) Post-default SAM mortality based on baseline Niger mortality adjusted for duration of and hazard ratio for untreated severe/moderate wasting
(e) Life table mortality based on age-specific regional mortality for non-wasted children from the 'Mortality and global health estimates' of the WHO Global Health Observatory Data Repository
(f) Mortality after transfer to hospital based on parent trial data
Note: Untreated SAM mortality based on Niger baseline mortality with adjustment for untreated severe wasting (hazard ratio=11.6)
