# Supplementary material for: Cost-effectiveness of routine versus indicated antibiotic therapy in the management of severe wasting in children
Source: Cost Eff Resour Alloc. 2022 Aug 3;20:38. doi: 10.1186/s12962-022-00374-z (PMC9351197; doi:10.1186/s12962-022-00374-z)
Supplement: Supplementary file 2 — Additional file 2: Table S1. Costs of therapeutic food and medical supplies per child-outcome. Table S2. Costs of personnel, infrastructure and logistical support per fixed cost category. Table S3. One-way sensitivity analysis results. Table S4. Probabilistic sensitivity analysis results with varying levels of coverage for the routine and indicated treatment strategies. [file 12962_2022_374_MOESM2_ESM.docx]

**Additional file 2**

Table S1. Costs of therapeutic food and medical supplies per child-outcome

|  | Nutritional recovery | | | Non-response | | | Default | | | Transfer to inpatient care | | | Death | |
| --- | --- | --- | --- | --- | --- | --- | --- | --- | --- | --- | --- | --- | --- | --- |
|  | Routine | Indicated | Routine | | Indicated | Routine | | Indicated | Routine | | Indicated | Routine | | Indicated |
| N | 790 | 752 | 72 | | 64 | 14 | | 10 | 316 | | 368 | 7 | | 6 |
| Average weight (kg) | 7.8 | 7.7 | 7.1 | | 7.1 | 7.5 | | 7.3 | 6.3 | | 6.3 | 6.3 | | 6.2 |
| Length of stay (days) | 28.3 | 30.2 | 60.4 | | 60.0 | 24.3 | | 24.9 | 29.7 | | 28.9 | 28.9 | | 17.5 |
|  |  |  |  | |  |  | |  |  | |  |  | |  |
| **Therapeutic food** | **35.98** | **38.39** | **85.27** | | **84.72** | **30.89** | | **31.66** | **34.10** | | **33.37** | **24.49** | | **14.83** |
| RUTF | 35.98 | 38.39 | 80.03 | | 79.59 | 30.89 | | 31.66 | 29.45 | | 28.82 | 24.49 | | 14.83 |
| F75 | 0.00 | 0.00 | 1.86 | | 1.82 | 0.00 | | 0.00 | 1.65 | | 1.62 | 0.00 | | 0.00 |
| F100 | 0.00 | 0.00 | 3.37 | | 3.31 | 0.00 | | 0.00 | 2.99 | | 2.93 | 0.00 | | 0.00 |
|  |  |  |  | |  |  | |  |  | |  |  | |  |
| **Medical supplies & materials** | **8.15** | **8.06** | **29.34** | | **29.25** | **8.15** | | **8.06** | **29.34** | | **29.25** | **8.15** | | **8.06** |
| Systematic treatment^1^ | 1.01 | 0.91 | 1.01 | | 0.91 | 1.01 | | 0.91 | 1.01 | | 0.91 | 1.01 | | 0.91 |
| Other medication & materials^2^ | 7.14 | 7.14 | 28.33 | | 28.33 | 7.14 | | 7.14 | 28.33 | | 28.33 | 7.14 | | 7.14 |
|  |  |  |  | |  |  | |  |  | |  |  | |  |
| **Total per child outcome** | **44.13** | **46.45** | **114.61** | | **113.96** | **39.05** | | **39.71** | **63.44** | | **62.61** | **32.65** | | **22.89** |

^1^ Measles vaccination, albendazole, rapid malaria test, Vitamin A, folic acid, and 250mg amoxicillin in the routine antibiotic group.

^2^ Outpatient/hospital kits and non-systematic medication

Table S2. Costs of personnel, infrastructure and logistical support per fixed cost category

|  | Inpatient care | | Outpatient care | | Community-based screening | |
| --- | --- | --- | --- | --- | --- | --- |
|  | Quantity  (per 1 site) | Cost | Quantity  (per 10 sites) | Cost | Quantity  (per population of 100,000) | Cost |
| **Personnel** | **-** | **520,992.38** | **-** | **648,901.38** | **-** | **72,989.29** |
| Nurse supervisor (expatriate staff) | 1 | 53,640.72 | 5 | 268,203.62 | 1 | 53,640.72 |
| Doctor (expatriate staff) | 2 | 107,281.45 | - | - | - | - |
| Nurses | 17 | 94,665.63 | 14.3 | 79,519.12 | 1 | 5,569.57 |
| Nutrition Assistant | 24 | 101,582.63 | 14.3 | 60,441.67 | - | - |
| Hygienist | 10 | 28,217.40 | - | - | - | - |
| Nurse supervisor (national staff) | 1 | 8,964.64 | 14.3 | 128,015.09 | - | - |
| Doctor (national staff) | 7 | 83,903.06 | 5 | 59,930.76 | - | - |
| Receptionist | 1 | 3,096.42 | - | - | - | - |
| Guard | 1 | 2,821.74 | - | - | - | - |
| Cook | 1 | 3,096.42 | - | - | - | - |
| Driver | 1 | 4,657.12 | 10 | 46,571.19 | - | - |
| Lab technician | 2 | 11,137.13 | - | - | - | - |
| Community health workers | - | - | - | - | 200 | 7560.00 |
| Ministry of Health incentives | 1 | 17,928.00 | 1 | 6,219.92 | - | - |
|  |  |  |  |  |  |  |
| **Infrastructure and logistical support** | **-** | **168,489.43** | **-** | **390,665.80** | **-** | **0** |
| Vehicles^1^ | - | 15,892.35 | - | 158,923.51 | - | - |
| Non-medical equipment and  supplies^2^ | - | 10,5327.8 | - | 3,124.36 | - | - |
| Buildings^3^ | - | 38,367.29 | - | 25,930.31 | - | - |
| Transport and logistical support | - | 3,139.03 | - | 673.62 | - | - |

^1^ Includes vehicles, parts, gasoline and maintenance.

^2^ Includes soap, covers, bed sheets, mosquito nets, meals for caregivers, water jugs/taps, chlorination kits, generator/cold chain/sterilizer, stove to prepare milk, transport from France, and furniture.

^3^ Includes cost of buildings, building maintenance and warehouses.

Table S3. One-way sensitivity analysis results

| **Variable** | **Base case value** | **Sensitivity analysis range** | **ICER with parameter lower bound** | **ICER with parameter upper bound** |
| --- | --- | --- | --- | --- |
| *Population demographics* | | | | |
| Proportion of population under 5 years | 20.4% | 15.3-25.5% | 8.52 | 8.52 |
| Prevalence of severe wasting in children under 5 | 5.3% | 4.0-6.6% | 8.52 | 8.52 |
| Severe wasting incidence correction factor | 7.2 | 5.4-9.0 | 8.52 | 8.52 |
| Severe wasting treatment program point coverage | 19.6% | 14.8-24.5% | 8.52 | 8.52 |
| *Natural history* | | | | |
| Annual background mortality rate for non-wasted  children 1-5y in Niger | 2.2% | 1.7-2.8% | 8.54 | 8.50 |
| Hazard ratio of mortality among children with  untreated moderate wasting | 3.4 | 2.6-4.3 | 8.53 | 8.51 |
| Hazard ratio of mortality among children with  untreated severe wasting | 11.6 | 8.7-14.5 | 8.64 | 8.41 |
| Duration of untreated severe wasting episode (weeks) | 20.2 | 15.2-25.3 | 8.57 | 8.47 |
| *Routine treatment outcomes at 12 weeks* | | | | |
| Number recovered after severe wasting treatment | 733 | 550-920 | Indicated strategy dominant* | 61.65 |
| Number of non-responders to severe wasting treatment | 63 | 47-79 | 14.56 | 1.64 |
| Number defaulting from severe wasting treatment | 12 | 9-15 | 11.12 | 7.03 |
| Number transferring to inpatient care | 370 | 278-463 | 10.67 | 5.24 |
| Number of deaths after transfer to inpatient care  during treatment | 5 | 4-6 | 9.87 | 7.48 |
| Number of deaths during severe wasting treatment | 21 | 16-58 | 51.48 | 2.12 |
| Average days to recovery | 28.3 | 21.1-35.4 | 65.01 | Indicated antibiotic therapy dominant* |
| Average days to default | 24.3 | 18.2-30.4 | 9.23 | 7.81 |
| Average days to death | 28.9 | 21.7-36.1 | 9.50 | 7.54 |
| Average days to transfer | 24.8 | 18.6-31.0 | 23.33 | Indicated antibiotic therapy dominant* |
| *Indicated treatment outcomes at 12 weeks* | | | | |
| Number recovered after severe wasting treatment | 700 | 525-875 | 65.45 | 1.02 |
| Number of non-responders to severe wasting treatment | 47 | 35-59 | 3.46 | 13.02 |
| Number defaulting from severe wasting treatment | 9 | 7-11 | 7.44 | 10.07 |
| Number transferring to inpatient care | 427 | 320-534 | 5.28 | 10.09 |
| Number of deaths after transfer to inpatient care  during treatment | 5 | 4-6 | 7.49 | 9.87 |
| Number of deaths during severe wasting treatment | 17 | 13-21 | 5.68 | 23.31 |
| Average days to recovery | 30.2 | 22.7-37.8 | Indicated antibiotic therapy dominant* | 54.60 |
| Average days to default | 24.9 | 18.7-31.1 | 7.98 | 9.06 |
| Average days to death | 17.5 | 13.1-21.9 | 8.04 | 9.00 |
| Average days to transfer | 24.1 | 18.1-30.1 | Indicated antibiotic therapy dominant* | 25.05 |

*Indicated antibiotic therapy had higher LYS and lower costs compared to routine antibiotic therapy.

Table S4. Probabilistic sensitivity analysis results with varying levels of coverage for the routine and indicated treatment strategies

| **Routine coverage** | **Indicated coverage** | **Probability of indicated strategy being optimal** | **Probability of indicated being dominant** |
| --- | --- | --- | --- |
| *Equal coverage for both strategies* | | | |
| 19.6% | 19.6% | 78.7% | 35.9% |
| 25.0% | 25.0% | 78.6% | 36.9% |
| 50.0% | 50.0% | 79.6% | 36.0% |
| 75.0% | 75.0% | 82.2% | 35.8% |
| 100.0% | 100.0% | 81.3% | 38.5% |
| *Indicated strategy coverage higher by 5.4 percentage points* | | | |
| 19.6% | 25.0% | 100.0% | 10.7% |
| 25.0% | 30.4% | 100.0% | 14.6% |
| 50.0% | 55.4% | 100.0% | 27.1% |
| 75.0% | 80.4% | 100.0% | 35.6% |
| *Indicated strategy coverage higher by 10.0 percentage points* | | | |
| 19.6% | 29.6% | 100.0% | 1.6% |
| 25.0% | 35.0% | 100.0% | 4.1% |
| 50.0% | 60.0% | 100.0% | 16.9% |
| 75.0% | 85.0% | 100.0% | 23.3% |
